# Limits in Laparoscopic Partial Splenectomy in Children

**DOI:** 10.3390/children9050605

**Published:** 2022-04-24

**Authors:** Christian Tomuschat, Michail Aftzoglou, Johanna Hagens, Michael Boettcher, Konrad Reinshagen

**Affiliations:** 1 Department of Pediatric Surgery, University Medical Center Hamburg-Eppendorf (UKE), 20246 Hamburg, Germany; michail.aftzoglou@uke.de (M.A.); j.hagens@uke.de (J.H.); k.reinshagen@uke.de (K.R.); 2 Department of Paediatric Surgery, Mannheim University Medical Center (UMM), 68167 Mannheim, Germany; michael.boettcher@umm.de

**Keywords:** laparoscopic splenectomy, partial splenectomy, hereditary spherocytosis, splenic cysts

## Abstract

The aim of this paper is to assess the effectiveness and perioperative complications of splenic surgeries in children. In 41 splenectomies, an anterior abdominal laparoscopic approach was used, with 35 including a partial laparoscopic splenectomy. Of these, three needed a conversion to open. Six patients had a total splenectomy, three of which were open. Patients ranged in age from 5 to 18 years. Splenectomy was performed for a variety of causes, including hereditary spherocytosis (*n* = 20), splenic cysts (*n* = 13), sickle cell disease (*n* = 3), primary malignancy (*n* = 1), sepsis (*n* = 1), embolism (*n* = 1), anemia (*n* = 1), and hypersplenism (*n* = 1). The average length of stay was 7.6 days, and the average operation time was 169.3 min. Pleural effusion in the left hemithorax was found in 31.6% of the patients, with 5.3% requiring a thorax drain. The majority of patients had the highest platelet count two weeks after surgery. There was no evidence of wound infection, pancreatic leak, colon perforation, or postoperative sepsis. The most encountered perioperative complication was bleeding with the need of transfusion (*n* = 6), and one patient needed a diaphragm repair. A partial splenectomy (PS) can be a difficult procedure with a steep learning curve. For most children who require a splenic operation, this should be the primary procedure of choice.

## 1. Introduction

Laparoscopic splenectomy (LS) was first described in adults in 1991 and then in children in 1993 [1]. Numerous reports involving various approaches followed [2,3]. Reduced pain medication use, shorter length of stay (LOS), fewer complications, and enhanced cosmesis are among the documented benefits of LS [1]. Concerns have been expressed about overlooked accessory spleens, splenomegaly treatment, and hemorrhage. Furthermore, the use of splenectomy in children is complicated due to the risk of post-splenectomy sepsis. When compared to children who have not had a splenectomy, the risk of overwhelming post-splenectomy sepsis (OPSI) in children under the age of five is elevated by a factor of 60 to 100 [4]. Although vaccines against Streptococcus pneumoniae, Meningococcus, and Haemophilus influenzae, as well as postoperative antibiotic prophylaxis, minimize the chance of OPSI, it is never eradicated. A PS is advocated as an alternative to total splenectomy for children with congenital hemolytic anemias, with the goal of removing enough spleen to provide the desired hematologic result while keeping splenic immune function [5,6,7]. However, it is impossible to conclude from the literature the amount by which this risk is lowered by exposing the child to a partial splenectomy versus a total splenectomy because there are no trials large enough or with a long enough follow-up [7]. Therefore, due to technical challenges and concerns about splenic regrowth, the use of partial splenectomy should be carefully balanced against the risks and benefits provided by LS. When planning a laparoscopic PS, it is important to consider the spleen’s segmental vascular supply, as the splenic artery splits into two (86%) or three (12%) lobar arteries in the hilum. However, about 2% of spleens have numerous lobar arteries or a single, undivided artery, making laparoscopic PS exceedingly difficult [8]. As a result, technical obstacles are common when doing a laparoscopic PS, especially in the beginning; nevertheless, when executing this procedure on a regular basis, a steep learning curve is usually experienced, and operation time, as well as complication rate, decreases over time [9]. Once the vascular pedicle has been determined, it is controlled by ligation of the terminal splenic arteries, which results in an ischemic demarcation of the parenchyma and allows its transection with minimal blood loss. A hemisplenectomy can be performed if two arteries are present. In the case of three arteries, either a one-third or a two-thirds splenectomy can be performed. Depending on the underlying pathology, either the upper or lower pole can be resected, and branches of the splenic artery to the lower or upper pole can be maintained. Aside from the vascular supply, coagulopathy, portal hypertension with hepatic cirrhosis, and splenic size greater than 27 cm can still complicate the laparoscopic approach and are considered relative contraindications to LS [10].

In the present series, we followed 41 children with hereditary spherocytosis (HS), sickle cell disease (SCD), and splenic cysts (SC) who had PSs to determine the partial splenectomy’s role in symptomatic children, and we carefully addressed the potential of an open approach in high-risk patients. Our findings indicate that a PS may be advantageous for a variety of congenital hemolytic anemias and splenic cysts.

## 2. Materials and Methods

### 2.1. Patients

The study followed the principles of the Helsinki Declaration and was approved by the local institutional review board, the Hamburg ethics committee (2022-300181-WF, 21 March 2022). From June 2012 to March 2022, the hospital records of all children who had their spleen partially or completely removed, either laparoscopically or open, at the University Medical Center Hamburg-Eppendorf (UKE) Pediatric Surgery Department were included in the study. Spleen removal owing to trauma which did not occur during the study period was an exclusion criterion. In total, 41 patients met the inclusion criteria.

### 2.2. Outcomes of the Study and Hypothesis

The study’s goal was to characterize the perioperative morbidity of partial splenectomy during childhood. The short-term results of laparoscopic PS, such as operation time, complications, conversion rate, intraoperative adverse events, and postoperative complication rate were the primary endpoints of our study. Splenic regrowth, LOS, and postoperative thrombocyte count were secondary outcomes.

### 2.3. Study Protocol

The medical patient records were analyzed with regard to the presentation, diagnostic studies, operative approach (laparoscopic, single-port, primary open, conversion to open), complications, functional results, splenic regrowth, and thrombocyte count. In addition, information on the length of hospital stay (LOS) was acquired. Patient demographics were calculated as median with interquartile range, as well as maximum and minimum values given as total range. Statistical analysis was performed descriptively using Microsoft Office Excel (version 16.56, Microsoft, Seattle, WA, USA) and GraphPadPrism (version 9.2.0, GraphPad, San Diego, CA, USA).

### 2.4. Description of Surgery

The patient is positioned in a supine, split-leg position (“French position”) with the left side tilted 45 degrees. To flex and support the patient, a tiny roll or vacuum mattress is employed. The surgeon stands between the legs of the operating table, while the assistant stands to the right. From the nipples to the symphysis, the abdomen is prepped and draped.

Through a subumbilical incision (10 mm), the fascia is opened, and a Gel Port combined with an Alexis Wound Protector (Applied Medical, Rancho Santa Margerita, CA, USA) is placed. This approach makes it easier to retrieve the specimen at the end of the procedure. A 10 mm camera port and a 5 mm instrument port are inserted via the GelSeal. CO_2_ insufflation is initiated at a pressure of 12 mmHg with a CO_2_ flow of 4–6 L/min, and a 5 mm camera (30 degree) is inserted. In the left lower quadrant, a 10 mm port is inserted. In the mid-epigastrium, an extra 5 mm port is placed. The vascular pedicle branches are carefully dissected and released for 1–2 cm in order to obtain sufficient and safe length. To gain access to the smaller sac, the gastrocolic ligament is divided. The short gastric veins are separated as needed, and the stomach is grasped sufficiently high on the posterior edge of the larger curvature for better exposure of the vascular pedicle and the anterior section of the splenic hilum. For dissection and sealing, we use the Maryland Ligasure (Medtronic, Dublin, IR). The vessels are clipped twice, once in the center and once at the periphery (Hem O Lok, Teleflex-Weck, Morrisville, NC, USA). Alternatively, the spleen’s distal end is sealed. Locking clips are recommended since the possibility of clip dislocation is reduced. After dividing the selected terminal splenic vessels, an ischemic demarcation line between the vascularized and ischemic regions can be seen. The spleen is then separated with a sealing device near the demarcation line but in the devascularized portion. The sealer is used to treat any remaining bleeding vessels. Upper pole resection is accomplished by incising the phrenosplenic ligaments and the upper lateral attachments in order to mobilize the upper pole. The posterior and splenorenal ligaments, as well as the lower lateral attachments, are split during a lower pole partial splenectomy. The resected spleen is carefully fragmented intraabdominally and retrieved piecemeal in a retrieval bag and can be delivered through the Gel Port. To guarantee proper hemostasis, the camera port is inserted once again. A drain may be implanted to detect postoperative bleeding early.

### 2.5. Follow-Up

Regular outpatient clinic visits were used to collect follow-up data. Patients with splenic cysts who received a PS are monitored every six months after they have been seen weekly to monitor the thrombocytes. There are also questions about satisfaction with functional and cosmetic results. In most circumstances, patients are seen weekly for a month before being seen only by a hematologist.

## 3. Results

### 3.1. Patient Database

**Figure 1:**
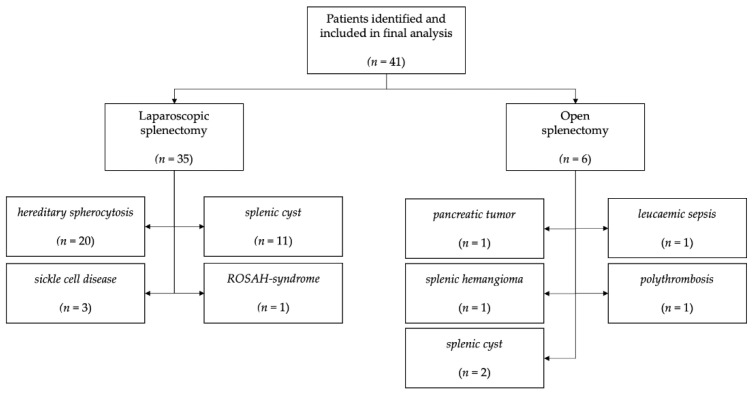
Patients that were operated and included in the study.

Between June 2012 and May 2021, our institution performed 41 splenectomies, 35 of which were laparoscopic and six of which were open. Out of the 41 children, 23 had hemolytic disorders, with 20 (87%) having HS and 3 (13%) SCD. A splenic cyst was discovered in thirteen children (Figure 1). The median age was 14.1 years, with the youngest child being 5.4 years old and the oldest 18.0 years. The BMI ranged from 14.0 to 29.0. The median longitudinal length of the spleen was 13.5 cm with the largest length being 23.3 cm (Table 1).

The median length of stay (LOS) in the laparoscopic operations group was 6.0 days, despite the fact that 71% of the patients had a five-day LOS; however, one patient stayed 55 days due to septic complications. Further study of the data revealed that, independent of the diagnosis or conversions, the duration of hospital stay was shorter over the last half of our time period.

In three cases, laparoscopic splenectomy was converted to an open procedure. The length of the spleen was greater than the sonographic findings in the first instance (23.3 cm), a 5-year-old had HS and an auxiliary spleen in the second case, and a cyst was in the upper portion of the spleen in the third case, making a laparoscopic approach very challenging.

The median age of those who had primary open splenectomies was 15.0 years (IQR 12.8–16.7 years), the median operation length was 159.0 min, and the median hospital stay was 7.5 days. The justification for open splenectomy was not based on the children’s somatometric features but rather on the underlying disease. One infant with sepsis was hospitalized for 55 days before succumbing to the underlying condition. In the second case, the spleen had a longitudinal length of 23.3 cm due to extensive thrombosis of the Vena portae hepatis causing extensive collaterals; the patient died as a result of the disease’s progression. In a third case, the spleen was excised en bloc with a solid pseudopapillary tumor of the pancreas tail. Finally, a splenic hemangioma was removed in the fourth. In the other two cases, partial open splenectomies were performed due to cysts, and, finally, sepsis as a consequence of leukemia necessitated a total spleen resection.

**Figure 2:**
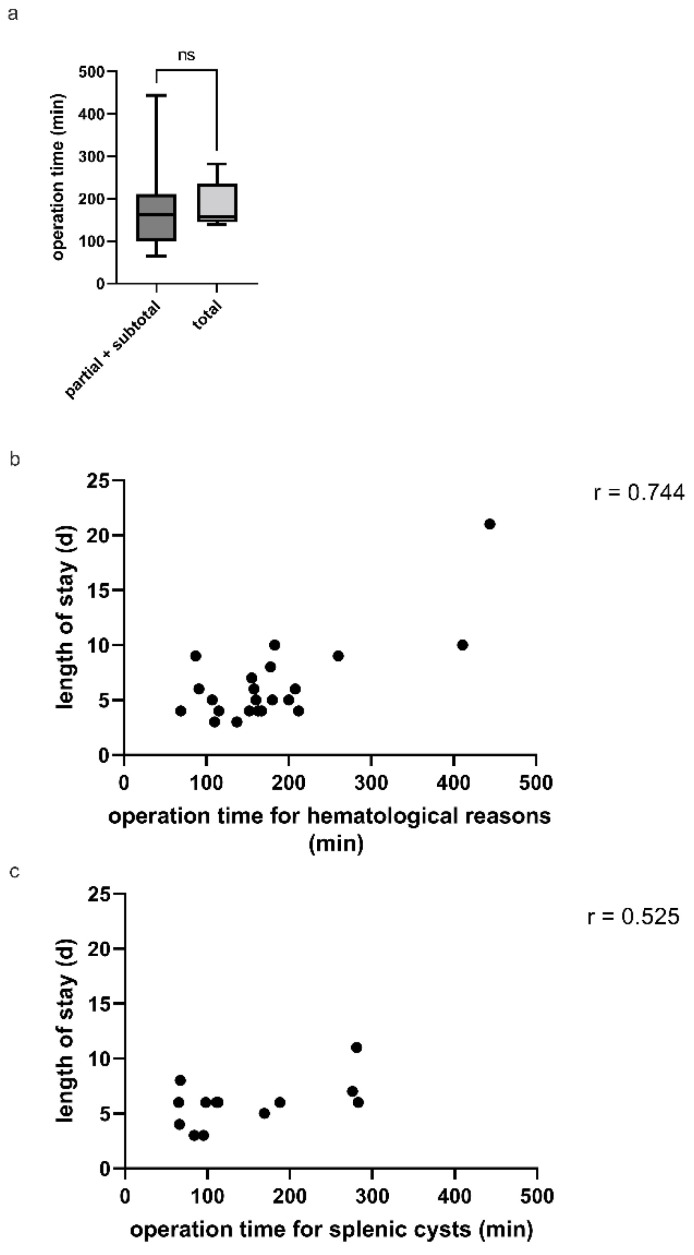
(**a**) Comparison of partial and total splenectomy operation times in minutes. (**b**) The correlation between the length of stay (days) and the operation time (minutes) is strong, with r = 0.744. (**c**) The operation time for splenic cysts in relation to the length of stay (days) demonstrates only a weak correlation with an r of 0.525.

Concomitant cholecystectomy was performed on seven children with hemolytic disease and cholelithiasis. Following the cholecystectomy, the patient’s bed was shifted to the right side, and a splenectomy was performed. The median splenic length was 14.5 cm (IQR 13.7–16.0), and the median age was 16.4 years. The LOS in these cases did not differ from those with singular splenectomy. The median operation time was 200.0 min compared to 159.0 min for the other laparoscopic cases (Figure 2, Table 2). It is worth noting that only one conversion was required. During these combined surgeries, no intraoperative problems were seen.

### 3.2. Perioperative Mortality and Morbidity

The intraoperative complications after both types of procedure were limited to spleen hemorrhage during excision and not during spleen vascular ligation. Transfusion was administered in six patients either during the operation or during the first postoperative hours and was based entirely on hemoglobin levels. Notably, a 14-year-old adolescent with HS was reoperated on the first postoperative day after undergoing a subtotal splenectomy to control the bleeding. In this case, 8.5 cm of the spleen was removed from a total length of 15.0 cm. Finally, in one patient with an upper pole cyst adhering to the diaphragm, a small portion of the diaphragm was removed, resulting in pneumothorax. The diaphragm was primarily laparoscopically repaired, and a chest drain was inserted. The surgical care went smoothly, and the chest drainage was removed on the fifth postoperative day.

A total of 38 patients were available for follow-up. Postoperatively, all patients were given antibiotics, with 90% of them receiving second-generation cephalosporines. All patients were subjected to a sonographic examination to locate the spleen and control for pleura effusion. The spleen was located in the left upper quadrant in all cases after partial splenectomy. On the third postoperative day, 31.6% of patients had a minor quantity of fluid in the left hemithorax, which resolved spontaneously without further treatment. No dyspnea or pneumonia were identified. There were also no postoperative cases of abscess, pancreatitis leakage, or colon perforation (Table 3).

### 3.3. Hematological Outcomes

**Figure 3:**
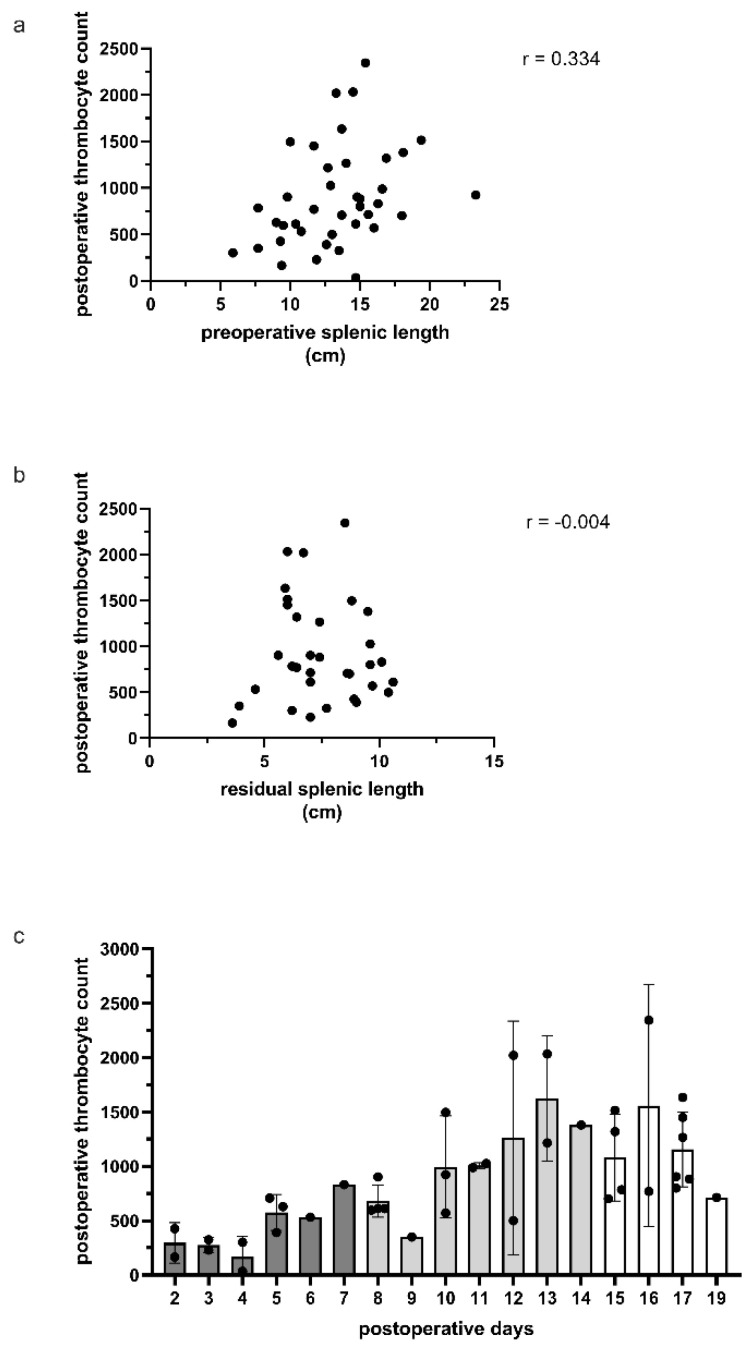
(**a**) The preoperative spleen size was related to the postoperative thrombocyte count, demonstrating a trend of higher thrombocytes postoperatively. (**b**) There was no association between residual spleen size and postoperative thrombocyte count. (**c**) The postoperative thrombocyte count was highest at approximately two weeks, followed by a slow fall after 17 days.

In all patients, a full blood count was obtained after surgery. During the first postoperative weeks, there were no increased bilirubin levels, but all patients developed temporary thrombocytosis. Due to their median age of 14.1 years, they were all treated subcutaneously with low-molecular heparin, with aspirin being administered only to those who had more than 1.000 × 10^3^ platelets. Only twelve cases exceeded that amount when the platelet count was documented. However, 24 of 39 available patients (61.5%) had more than 700 × 10^3^ platelets after surgery. Surprisingly, it was discovered that, in the majority of cases during follow-up, patients had the highest platelet count two weeks after the operation, whilst a normal platelet count was observed after the 20th postoperative day (Figure 3).

## 4. Discussion

The role of partial splenectomy in children with hematological diseases, such as HS and SCD, as well as surgical causes such splenic cysts, is currently being debated [11]. This is due to the likelihood of serious perioperative complications, such as hemorrhage, as well as the possibility of splenic regrowth [12]. Furthermore, due to anatomical reasons, such as splenic size or massive splenic cysts, a laparoscopic partial splenectomy might be a challenging task [13]. This was evident in our series, where the operating time ranged from 65 to 411 min compared to an open approach, which ranged from 67 to 287 min. One would think that the open technique would be faster; however, in our cohort, patients who were treated to an open approach were extremely critical ill children, and two of them died over the course of the disease or were subjected to a conversion from a primary laparoscopic approach. There are two reasons for the wide range of the length of the operation in the laparoscopic group. First, the group was treated during the period when the laparoscopic partial splenectomy was being established in our hospital; this is also reflected by the steep learning curve and a significant drop in the operation time in the last patients. Second, there were seven simultaneous cholecystectomies in that group, which slightly extended the operation time. Furthermore, PS is frequently performed by a senior surgeon, but the cholecystectomy in the same patient is frequently performed by the assistant surgeon. However, following a splenectomy, residual hemolysis increases the chance of gallstone development and symptomatic gallstones or cholecystitis; hence, cholecystectomy is recommended if gallstones are found [11]. During laparoscopy for PS, a simultaneous cholecystectomy should be performed and usually results in uncomplicated postoperative care and a minimal increase in operation time. Therefore, the presence of gallstones in adolescent patients with hemolytic splenomegaly must be evaluated preoperatively to avoid any unpleasant occurrence of cholecystitis and unnecessary laparoscopies in the future [14]. However, we clearly found that the length of the operation correlated strong with the LOS. According to some research, laparoscopic PS is more painful and takes longer to recover from than laparoscopic TS. The cause is unknown; however, it could be related to the prolonged operating time, which could result in pleural effusion or blood in the peritoneal cavity [15].

When looking at the perioperative complications, six out of 38 patients needed a blood transfusion postoperatively due to hemorrage; in fact, one patient needed a reoperation for hemostasis. Because the risk is obvious higher in patients selected for PS, preoperative patient optimization should result in better outcomes. Most often, the bleeding arises from the cut edge of the splenic remnant, as well as from the vascular supply to the remnant. Transfusions given before surgery to resolve preoperative anemia can shorten recovery time and hospital stay [16]. We also had 12 patients with pleural effusions, but only two of them needed a pleural drainage. One pneumothorax was related to the fact that the diaphragm was opened, resecting a huge splenic cyst. In that case, the diaphragm was primarily repaired. In total, we had thirteen cysts in our series, nine of which were treated laparoscopically, and two of which were treated open. After definitive therapy, none of them exhibited evidence of recurrence of the splenic cysts, which can be extremely difficult to treat due to their size and location [17]. Since simple deroofing and omentopexy proved to be inadequate, we recommend that partial or even total laparoscopic splenectomy should be the treatment of choice [18]. Due to the postoperative course, early mobilization may also prevent lung infections, also emphasizing the need to reduce surgical trauma and for the use of laparoscopy. Postoperative complications, such as pleura effusion or increased thrombocytes with potential thrombotic events, should always be considered; however, intervention is not required in all cases [3]. However, vascular thrombosis is a major possible consequence after TS or PS, and it is identified clinically in up to 10% of patients [3]. This includes portal vein thrombosis, deep vein thrombosis, and pulmonary embolism; symptomatic portal vein thrombosis occurs in 1.6% of children, and 11% of children are asymptomatic [19]. Therefore, platelet levels should be monitored postoperatively and in the outpatient clinic department, since the highest levels are found around two weeks after splenectomy, in our experience. However, aspirin therapy appears to be sufficient to prevent any thrombotic events. In cases of a large platelet count increase, a functional test (ROTA) could access and stratify the risk of thrombosis, indicating the length of therapy and aspirin dose required. This could be extremely beneficial for more complicated patients with several comorbidities. However, no correlation was discovered when comparing preoperative splenic size to postoperative thrombocyte count; we hypothesized that, as splenic length increased preoperatively, the postoperative rebound of thrombocytes would exhibit a stronger correlation, since the spleen is the main site removing platelets from the blood stream [4]. The aim was to identify patients who were at high risk of developing thrombotic events after surgery and were good candidates for starting antithrombotic medication. However, there was also no correlation between residual splenic length and postoperative thrombocyte count. It is unknown if the splenic remnant retains immune competence. The significance of PS derives from its advantages in keeping, in part, the immune splenic function and, at the same time, the decrease in hemolysis in those patients [20]. However, the theoretical advantage of this strategy may be the prevention of overwhelming post-splenectomy infection (OPSI) from encapsulated organisms and other long-term asplenia consequences such as thrombotic events [11]. However, this idea remains unsubstantiated because the incidence of OPSI has become extremely low in the age of routine immunization and prophylactic antibiotic medication [4].

There is no apparent association between the extent of splenic regrowth or splenosis and immunological protection, and preliminary data aggravate this danger by showing that immune protection reduces with age independent of type of splenectomy [11]. Postoperative MRT imaging provides the best evidence of splenic regrowth, although sonography appears to be sufficient for early postoperative follow-up. Nonetheless, the problem of regrowth is common, but, even if it occurs, anemia must be severe to necessitate removal of the remnant spleen [15]. Splenic regrowth was uncommon in our group, and only one patient required a reoperation for hematologically significant regrowth resulting in anemia.

However, an auxiliary spleen, which is essential to detect for hemolytic patients, can be missed during sonography.

## 5. Conclusions

The surgical technique of choice for splenectomy is determined by the primary indication. In most circumstances, an elective laparoscopic complete or partial splenectomy is possible [21]. A growing body of literature and advancements in hemostatic equipment imply that it may be the therapy of choice for patients with hemolytic disorders or splenic cysts, allowing adequate excision of the required splenic mass while maintaining healthy splenic tissue and limiting collateral damage. However, increased length of the operation time may outweigh the possible benefits of laparoscopy, so patients should be carefully selected before being submitted to a PS.

## 6. Limitations

The current study may be limited in accuracy due to missing data in the charts due to the study methodology. Furthermore, the small sample size and the study’s single-center, retrospective nature limits the conclusions derived from the current study.

## Figures and Tables

**Table 1:** Patient characteristics (total *n* = 41).

	*n* (%)	Median	IQR	Range
male	20 (48.8)			
female	21 (51.2)			
age at surgery (y)		14.1	(11.1–16.6)	(5.4–18.0)
mortality	2 (4.9)			
BMI *		20.0	(16.5–21.3)	(14.0–29.0)
height (cm) *		158.0	(142.0–172.0)	(110.0–184.0)
weight (kg)		50.0	(37.4–57.6)	(20.0–82.3)
*main diagnosis*				
hereditary spherocytosis	20 (48.8)			
splenic cyst	13 (31.7)			
sickle cell anemia	3 (7.3)			
other	5 (12.2)			

y = years, cm = centimeters, kg = kilogram, IQR = interquartile range. Total ranges given for median values. Median age of the study patients matches mean age at operation. * BMI and height were only available for *n* = 31 patients.

**Table 2:** Surgical data (total *n* = 41).

	*n* (%)	Median	IQR	Range
surgical procedures				
laparoscopic splenectomy	35 (85.4)			
open splenectomy	6 (14.6)			
partial or subtotal splenectomy	36 (87.8)			
total splenectomy	5 (12.2)			
singular splenectomy	34 (82.9)			
concomitant cholecystectomy	7 (17.1)			
preoperative splenic length (cm)		13.5	(10.2–15.5)	(5.9–23.3)
length of residual spleen (cm) *		7.4	(6.2–9.4)	(3.6–14.0)
operation time (min) **		160.0	(109.0–210.0)	(65.0–444.0)
laparoscopic splenectomy		160.0	(107.0–212.0)	(65.0–444.0)
open splenectomy		159.0	(122.0–212.0)	(67.0–282.0)
concomitant cholecystectomy		200.0	(137.0–260.0)	(107.0–444.0)
total length of hospital stay (d)		6.0	(4.0–8.0)	(3.0–55.0)
hereditary anemia		5.0	(4.0–8.0)	(3.0–21.0)
splenic cyst		6.0	(4.5–6.9)	(3.0–11.0)
laparoscopic splenectomy		6.0	(4.0–7.0)	(3.0–23.0)
open splenectomy		7.5	(5.0–21.3)	(5.0–55.0)

d = days, min = minutes, cm = centimeters, IQR = interquartile range. Total ranges given for median values. * Length of residual spleen included all partial and subtotal resections except one (*n* = 35). ** Operation time of laparoscopic splenectomy also included concomitant cholecystectomies. Operation time of open splenectomies included all primary open splenectomies, not including conversions from laparoscopy. All performed concomitant cholecystectomies were laparoscopic procedures.

**Table 3:** Perioperative complications (patients *n* = 41).

	*n*
pleural effusion	12
need for pleural drainage	2
need for blood transfusion	6
postoperative bleeding	2
pneumothorax	1
sepsis	0
wound infection	0
pancreatic leak	0
colon perforation	0
death *	2
**total**	**23**

* both patients died due to complications of the underlying diseases unrelated to the surgical procedure.

## Data Availability

The data presented in this study are available on request from the corresponding author. The data are not publicly available due to privacy concerns.
